# Functional Properties Modulation of Soybean, Adzuki Bean, Mung Bean, and Peanut Proteins Through Alkaline Extraction pH

**DOI:** 10.1002/fsn3.71140

**Published:** 2025-11-05

**Authors:** Hyun‐Jin Park, You‐Geun Oh, Areum Chun, Jung Hyun Seo, Eunyoung Oh, Ji Ho Chu, Young Kwang Ju, Sang‐Jin Ye

**Affiliations:** ^1^ Crop Post–Harvest Technology Division, Department of Central Area Crop Science National Institute of Crop Science (NICS), Rural Development Administration (RDA) Suwon Republic of Korea; ^2^ Paddy Crop Research Division, Department of Southern Area Crop Science National Institute of Crop Science (NICS), Rural Development Administration (RDA) Miryang Republic of Korea

**Keywords:** adzuki bean protein, alkaline extraction, functional properties, mung bean protein, peanut protein, soybean protein

## Abstract

This study investigates the effects of alkaline extraction pH on the yield, composition, structure, and functional properties of proteins from soybeans, adzuki beans, mung beans, and peanuts. Protein extraction at highly alkaline conditions (pH 12) increased protein yield by 10.88% in adzuki beans and by 11.65% in mung beans but resulted in remarkably darker coloration. The highly alkaline conditions decreased the particle size of the soybean proteins by 23.96% and adzuki bean proteins by 23.2%, and altered the amino acid compositions of adzuki bean proteins. Depending on the extraction conditions, differences were observed in the secondary and tertiary structures as well as in the subunit composition of proteins, and these effects were distinct among the different legumes. The functional properties were notably affected, with protein solubility indices decreasing by 10.34% at high pH. Moreover, the water solubility index showed a remarkable decrease in adzuki and mung beans by 64.29% and 43.80%, respectively. The foaming capacity was higher for proteins extracted at pH 12, suggesting their suitability for applications requiring a soft and voluminous texture, whereas the emulsifying activity was higher at pH 9, which suggests suitability for emulsified products. Thus, adjusting the extraction pH may effectively modulate the functionality of legume proteins for food applications.

## Graphical abstract

This study investigates the effects of alkaline extraction pH on the yield, composition, and functional properties of proteins from soybeans, adzuki beans, mung beans, and peanuts.

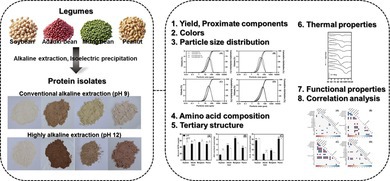

## 1 Introduction

Proteins are classified as animal‐ and plant‐based proteins depending on their source. Animal proteins have diverse molecular structures that make them suitable for food processing (McClements and Grossmann 2021). However, animal‐based proteins raise concerns regarding health issues, such as chronic diseases and allergies, as well as ethical considerations for animal welfare and environmental sustainability (Avelar et al. 2021). Consequently, consumer interest in plant‐based proteins has increased, driven by the growth of the plant‐based alternative food market, which aims to replicate the texture and flavor of animal‐based foods using various plant‐derived proteins (Bakhsh et al. 2021). However, plant storage proteins are primarily globular and exhibit inferior functional properties in food processing compared with animal proteins (McClements and Grossmann 2021). Thus, various processing techniques have been developed to enhance the functional properties of plant proteins (Ye et al. 2024).

Intrinsic factors that affect protein functionality include composition and structure, whereas extrinsic factors include temperature and extraction conditions (Eze et al. 2022). Protein extraction is the first critical step in producing plant‐based alternative foods. By applying appropriate extraction methods, the desired functional properties of alternative foods can be attained without requiring additional processing methods (Huang et al. 2024). Among the various methods, alkaline extraction is the most widely used technique for protein recovery, which involves dissolving proteins at pH 8–11 and precipitating them near their isoelectric point, typically at pH 4.0–4.8 (Eze et al. 2022). Alkaline conditions modify the secondary, tertiary, and quaternary structures of proteins, exposing hydrophobic regions and free sulfhydryl groups to the exterior and subsequently affecting protein functionality (Momen et al. 2021). Additionally, alkaline conditions increase the reactivity of specific amino acid residues, such as cysteine, leading to protein polymerization through thiol‐disulfide exchange reactions or protein aggregation via hydrophobic and disulfide bonds (Ruiz et al. 2016). Wu et al. (2021) reported that increasing the extraction pH for tartary buckwheat proteins increased the ratio of α‐helices and random coils, decreased the ratio of β‐sheets, and improved emulsifying properties. Similarly, Gao et al. (2020) reported that the extraction pH affects the sulfhydryl group content in pea proteins, with higher pH levels leading to reduced protein solubility.

Soybeans ( *Glycine max* L.) are a representative source of plant‐based proteins, with a high protein content of 40% (Preece et al. 2017). Due to their high nutritional value, they are often considered a viable meat substitute, especially for individuals with cardiovascular diseases (Bakhsh et al. 2021). Adzuki beans ( *Vigna angularis* L.) contain 21.6% protein (Guo et al. 2025; RDA 2018). These beans exhibit high protein digestibility (91%) and superior solubility under acidic conditions, making them suitable for acidic beverages. However, studies examining their applications in plant‐based alternative foods are limited (Li et al. 2022). Moreover, mung beans ( *Vigna radiata* L.) contain 25% protein (Du et al. 2018). Compared with soybean proteins, mung bean proteins provide higher thermal stability and additional benefits, including antihypertensive, antioxidant, and anti‐aging effects (Huang et al. 2024). Although the use of mung bean protein in alternative foods is currently less common than the use of soybean proteins, research in this area is gradually increasing (Huang et al. 2024). Peanuts ( *Arachis hypogaea* L.) contain 25.7% protein, with a lower content of the nutritional inhibitor trypsin, which comprises approximately 20% of soybean proteins, making it easier to digest and absorb. Additionally, peanut proteins have a milder flavor than soybeans, making them promising candidates for use in plant‐based alternative foods (Cui et al. 2023; Wang et al. 2014).

Seonpung, Arari, Dahyun, and Sinpalkwang are the primary cultivars of soybeans, adzuki and mung beans, and peanuts, respectively, grown in South Korea. Seonpung is known for its high isoflavone content and suitability for tofu production (Kim et al. 2017), whereas Arari is recognized for its excellent antioxidant properties and suitability for paste production (Desta et al. 2022). Research on Dahyun has primarily focused on its sprout‐processing characteristics and starch properties (Chu et al. 2023; Oh et al. 2024). Sinpalkwang is rich in linolenic acid; its defatted powder extract and kernels have demonstrated α‐glucosidase inhibitory activity (Ha et al. 2020; Kim et al. 2019; Pae et al. 2016). However, the functional properties of these proteins have not been studied.

Therefore, this study aimed to provide foundational data for the development of alternative food ingredients by extracting proteins from major cultivars of soybeans, adzuki beans, mung beans, and peanuts grown in Korea under highly alkaline conditions. This research extends traditional protein extraction methods and evaluates the functional properties of the extracted proteins.

## 2 Material and Methods

### 2.1 Plant Material and Sample Preparation

The experimental materials used in this study included soybeans (cv. Seonpung), adzuki (cv. Arari) and mung beans (cv. Dahyun), and peanuts (cv. Sinpalkwang). These materials were harvested in 2023 from the experimental fields of the Southern Crop Division of the National Institute of Crop Science, Korea. The samples were ground using a grinder (FM–700, Hanil Inc., Wonju, Korea) and passed through a 40‐mesh sieve for experimental analysis.

Soybeans and peanuts were defatted prior to protein extraction due to their high fat contents (~20% and ~40%), whereas adzuki beans and mung beans, with much lower fat levels (0.5%–1.5%), were extracted without defatting. The defatting process involved the addition of n‐hexane at a ratio of 10 times the sample weight (w/w), stirring at room temperature for 24 h, and drying the precipitate for 24 h before use. Protein extraction was conducted by adding distilled water (adjusted to pH 9 or 12 using 1 M NaOH) to 100 g of the sample at a ratio of 10 times the sample weight and stirring for 2 h. The supernatant containing the extracted protein was then adjusted to pH 4.5 using 1 M HCl, stirred for 30 min (to precipitate the protein), neutralized, and subsequently freeze‐dried. The dried proteins were ground and passed through a 100‐mesh sieve for further analysis. The extraction yield is expressed as the ratio of the protein extracted using the above process to the crude protein content in the raw material.

### 2.2 Proximate Composition

The crude protein content was determined by the Dumas method using an automated TruMac nitrogen/protein analyzer (Leco, Michigan, USA) and was calculated using a nitrogen conversion factor of 6.25. Crude fat content was determined by the soxhlet extraction method using n‐hexane. For analyzing ash content, 1 g of ground sample was ashed at 550°C for 3 h in an electric muffle furnace (DS–84E, Dasol Scientific, Hwaseong, Korea), and the weight was measured before and after ashing. Crude carbohydrate content was calculated by difference, subtracting the measured percentages of crude protein, fat and ash from 100%.

### 2.3 Color and Total Polyphenol Contents

Color measurements were performed using a colorimeter (CM3700A; Minolta, Osaka, Japan). Samples were placed in transparent plastic containers and sealed with Parafilm, and lightness (L*), redness (a*), and yellowness (b*) values were recorded. The color values of the standard white plate used were L* = 98.81, a* = −0.09, and b* = −0.37.

Total polyphenol contents were measured using the method described by Velioglu et al. (1998). Briefly, 10 μL of extract was mixed with 200 μL of Na_2_CO_3_, left for 3 min, and then 10 μL of Folin–Ciocalteu's phenol reagent was added. After 27 min, absorbance was recorded at 750 nm using a microplate reader (Varioskan LUX, Thermo, Massachusetts, USA). Gallic acid was used for calibration, and results were expressed as mg gallic acid equivalents (GAE) per g protein sample.

### 2.4 Particle Size Distribution

Particle size was measured by dispersing the sample in ethanol at a concentration of 0.03% using a particle size analyzer (Mastersizer 2000, Malvern Inc., Malvern, England). The particle diameters corresponding to 10%, 50%, and 90% of the total particle volume, referred to as *d*(0.1), *d*(0.5), and *d*(0.9), respectively, along with the volume mean diameter (*D*[4,3]), was also analyzed.

### 2.5 Amino Acid Composition and In Vitro Protein Digestibility

A protein sample (0.5 g) was placed in a Pyrex tube, followed by the addition of 10 mL of 6 N HCl. After purging the tube with N_2_ gas for a few seconds to create an inert atmosphere, the tube was sealed and hydrolyzed at 110°C in a high‐temperature thermostat for 24 h. After hydrolysis, the Pyrex tube was removed and cooled at room temperature. The cooled sample was filtered through Whatman No. 2 filter paper, and distilled water was added to adjust the final volume to 100 mL. A portion of the supernatant was collected, purified using a Sep‐Pak C18 cartridge to remove impurities, and prepared for amino acid analysis. Amino acid analysis was performed using an L‐8080 automatic amino acid analyzer (Hitachi, Tokyo, Japan). The mobile phases used for the analysis were PH1, PH2, PH3, PH4, PH–RG, R–3, C–1, ninhydrin solution, and buffer solution (Fujifilm Wako Pure Chemical, Osaka, Japan). An ion‐exchange column (#2622SC PF; Hitachi, Tokyo, Japan) was used. During the analysis, the column temperature was maintained at 50°C, and the reaction chamber temperature was set at 135°C. A standard amino acid mixture (Ajinomoto; Takara, Kusatsu, Japan) was used for calibration.

The in vitro protein digestibility (IVPD) of proteins was assessed using the K‐PDCAAS kit (Megazyme, Bray, Ireland) in accordance with the manufacturer's instructions. Approximately 500 mg of samples were sequentially digested with pepsin, trypsin, and chymotrypsin, and the reactions were terminated by heating, followed by trichloroacetic acid precipitation and centrifugation. Supernatants, along with calibration standards, blanks, and casein (positive control), were incubated in a hot‐air shaking incubator (70°C, 35 min, 300 rpm), and primary amines were quantified spectrophotometrically at 570 nm after ninhydrin reaction using L‐glycine as a standard. IVPD values were calculated relative to casein.

### 2.6 Protein Structural Analysis

#### 2.6.1 ATR‐FTIR Spectroscopy

ATR‐FTIR spectra were collected on a Spectrum 3 FT‐IR spectrometer (PerkinElmer, Waltham, MA, USA) equipped with the UATR accessory using a diamond/KRS‐5 crystal. Spectra were acquired in absorbance mode over 4000–400 cm^−1^ at a spectral resolution of 4 cm^−1^, with 32 co‐added scans per sample.

#### 2.6.2 Tertiary Structure

Surface hydrophobicity was measured using the method described by Ngo and Shahidi (2021). A 1‐mL sample (1 mg/mL) was mixed with 200 μL of a bound bromophenol blue solution and incubated at room temperature for 10 min. After incubation, the mixture was centrifuged at 3000 *g* for 15 min; the supernatant was diluted with buffer. The absorbance was measured at 595 nm using a spectrophotometer (Evolution 600, Thermo, Massachusetts, USA).

The sulfhydryl group and disulfide bond contents were determined according to the methods described by Gong et al. (2016). For the disulfide bond content measurements, 120 mg of the sample was mixed with 10 mL of Tris/glycine buffer containing 8 M urea and stirred for 30 min. The supernatant was collected for analysis after centrifugation at 10,000 *g* for 30 min. The sulfhydryl group content was measured by mixing 2 mL of the supernatant with Ellman's reagent and recording the absorbance at 412 nm. For disulfide bond content measurement, 4 μL of β‐mercaptoethanol was added to 2 mL of the supernatant, and the mixture was incubated at room temperature for 2 h. After incubation, 12% TCA was added, and the mixture was allowed to react for 1 h before being centrifuged at 3000 *g* for 10 min. The resulting precipitate was dissolved in buffer and mixed with Ellman's reagent; the absorbance at 412 nm was measured.

#### 2.6.3 SDS‐PAGE

Sulfate–polyacrylamide gel electrophoresis (SDS‐PAGE) was performed to analyze protein molecular weight patterns using Any kD Mini‐Protean TGX Precast gels (Bio‐Rad, Contra Costa, USA). Samples were mixed with 5× sample buffer (62.5 mM Tris–HCl, pH 6.8, 10% glycerol, 1% LDS, 0.005% bromophenol blue) and adjusted to a final protein concentration of 2 mg/mL prior to loading. Electrophoresis was conducted at 250 mA, and gels were subsequently stained with Coomassie blue solution (Bio‐Rad) to compare protein molecular weight distributions.

### 2.7 Thermal Properties Analysis

Thermal properties were analyzed using a differential scanning calorimeter (DSC Q1000, TA Instruments Inc., Delaware, USA). The instrument temperature was increased from 20°C to 160°C at a rate of 7°C/min. A 9‐mg protein sample was sealed in a stainless‐steel DSC pan for measurement. The onset temperature (*T*_o_), peak temperature (*T*_p_), and conclusion temperature (*T*_c_) were determined from the endothermic curve, and the denaturation enthalpy (ΔH) was calculated from the endothermic peak area.

### 2.8 Functional Properties

The protein solubility was determined using the Bradford method. The sample was reacted with bicinchoninic acid for 10 min, and the absorbance was measured at 595 nm using a microplate reader (Varioskan LUX, Thermo, Massachusetts, USA). The protein concentration was calculated based on a standard curve prepared using bovine serum albumin. The water solubility index was obtained by mixing 1 g of the sample with 40 mL of distilled water, stirring at room temperature for 1 h, and centrifuging at 3000 *g* for 20 min. The supernatant was then dried in a drying oven (DS–80–2; Dasol Scientific, Hwaseong, Korea) for 24 h, and the weight of the dried residue was divided by the initial sample weight.

The water and oil absorption capacities were determined by mixing 1 g of the sample with 40 mL of distilled water or soybean oil. The mixture was stirred at room temperature for 1 h and centrifuged at 3000 *g* for 20 min. The weight of the precipitate was measured, and the absorbed water or oil was expressed as the weight of water or oil absorbed per gram of the sample. Foaming capacity was evaluated by dispersing 2.5 g of the sample in 50 mL of distilled water and homogenizing the mixture at speed 4 for 1 min and 30 s using a homogenizer (HG–15A, Daihan Science, Wonju, Korea). The foam volume was measured using a graduated cylinder and divided by the initial liquid volume to calculate the foaming capacity. The foam stability was determined by measuring the foam volume 30 min after formation and dividing it by the initial foam volume.

The emulsifying activity and stability indices were measured by mixing a 0.2% protein solution and soybean oil in a 3:1 ratio and homogenizing the mixture at a speed of 4 for 1 min. The resulting emulsion was diluted and mixed with 0.1% SDS; absorbance at 500 nm was recorded. The EAI and ESI were calculated using the formula described by Xia et al. (2021).

### 2.9 Statistical Analysis

Statistical analyses were performed using the agricolae package (version 1.3‐7) in the R software (version 4.4.1). Two‐way analysis of variance (ANOVA) was conducted to analyze differences based on the legume type and the extraction pH, followed by Tukey's post–hoc test to identify significant differences at *p* < 0.05. An independent *t*‐test was used to compare differences in the extraction pH within the same legume type. All experiments were conducted at least three times. Pearson correlations were used to analyze potential relationships among properties.

## 3 Results and Discussion

### 3.1 Protein Extraction Yield and Proximate Composition

The results of the protein yield and proximate composition analyses at different extraction pH levels are presented in Table 1. A two‐way ANOVA revealed significant interaction effects between the legume type and extraction pH for protein yield (*p* < 0.01), crude protein (*p* < 0.001), crude ash (*p* < 0.001), and crude carbohydrates (*p* < 0.001). The protein yield ranged from 58.87% to 79.18% at pH 9 and from 59.48% to 79.57% at pH 12, with peanuts exhibiting a higher protein yield (79.18%–79.57%) than other legumes (58.87%–76.10%). Within the same legume type, the protein yield was higher at pH 12 than at pH 9 for adzuki and mung beans. Crude protein content ranged from 79.02% to 96.54% at pH 9 and from 80.12% to 94.32% at pH 12, with peanuts exhibiting a higher crude protein content (94.32%–96.54%) than other legumes (79.02%–87.76%). Additionally, within the same legume type, the crude protein contents of soybeans, adzuki beans, and peanuts were higher at pH 9. Conversely, mung beans exhibited higher crude protein content at pH 12; however, the differences were marginal. The ash content ranged from 2.67% to 5.61% at pH 9 and from 2.13% to 9.83% at pH 12. Among the legumes, mung bean proteins exhibited the highest ash content (5.61%–9.83%), whereas peanut proteins had the lowest ash content (2.13%–2.67%). Within the same legume type, the ash content of proteins extracted from soybeans and mung beans was higher at pH 12, whereas that of proteins extracted from peanuts was slightly higher at pH 9. The crude carbohydrate content ranged from 0.48% to 15.19% at pH 9 and from 3.22% to 14.06% at pH 12. Among the legumes, peanut proteins had the lowest carbohydrate content (0.48%–3.22%). Within the same legume type, the crude carbohydrate content of proteins extracted from mung beans was higher at pH 9, whereas that of proteins extracted from peanuts was higher at pH 12.

**TABLE 1:** Effect of the extraction pH on extraction yield and proximate components of different protein sources.

Legumes	Extraction pH	Protein yield (%)	Crude protein (%)	Crude fat (%)	Crude ash (%)	Crude carbohydrate (%)
Soybean	pH 9	60.69 ± 3.33^a^	87.76 ± 0.11**	0.32 ± 0.01	3.67 ± 0.04	8.28 ± 0.02
	pH 12	59.48 ± 0.18	85.91 ± 0.08	0.34 ± 0.01	4.00 ± 0.01**	9.85 ± 0.05
Adzuki bean	pH 9	68.63 ± 0.76	83.28 ± 0.31*	0.31 ± 0.00	4.71 ± 0.04	11.70 ± 0.05
	pH 12	76.10 ± 0.69**	80.12 ± 0.56	0.30 ± 0.01	5.52 ± 0.39	14.06 ± 1.06
Mung bean	pH 9	58.87 ± 1.72	79.02 ± 0.06	0.28 ± 0.01	5.61 ± 1.19	15.19 ± 1.05**
	pH 12	65.73 ± 0.53*	80.42 ± 0.02***	0.29 ± 0.01	9.83 ± 0.14**	9.47 ± 0.27
Peanut	pH 9	79.18 ± 1.87	96.54 ± 0.11*	0.31 ± 0.01	2.67 ± 0.01***	0.48 ± 0.02
	pH 12	79.57 ± 0.64	94.32 ± 0.57	0.33 ± 0.02	2.13 ± 0.01	3.22 ± 0.05**
Legumes (L)		***^b^	***	ns	***	***
pH (P)		**	***	ns	***	**
L × *P*		**	***	ns	***	***

^a^ Significant differences between extraction conditions within the same protein source were determined using a *t*‐test (*p* < 0.05, *p* < 0.01, and *p* < 0.001 indicated by *, **, and ***, respectively).

^b^ Significant differences between the legume type and the extraction pH were determined using a two‐way ANOVA test (*p* < 0.05, *p* < 0.01, and *p* < 0.001 indicated by *, **, and ***, respectively).

In this study, protein yield tended to increase under high‐alkaline extraction conditions, whereas crude protein and ash content exhibited different trends depending on the legume type. Moreover, alkaline extraction appeared to be most suitable for peanuts, as peanut proteins exhibited a higher protein yield and crude protein content and a lower crude ash and carbohydrate content than other legumes. Momen et al. (2021) reported that high pH conditions improved protein yield in castor beans and lentils during protein extraction. This was attributed to the disruption of hydrogen bonds between lignin and cellulose, major cell wall components, under highly alkaline conditions. Conversely, the lower crude protein content of soybeans, adzuki beans, and peanuts under highly alkaline conditions may be attributed to the simultaneous extraction of starch and other polysaccharides. Furthermore, the high ash content of soybean and mung bean proteins may have resulted from the formation of salts during protein extraction, owing to the use of strong acids and alkalis for pH adjustment (Weemaes et al. 1998).

### 3.2 Color and Total Polyphenol Contents

**FIGURE 1:**
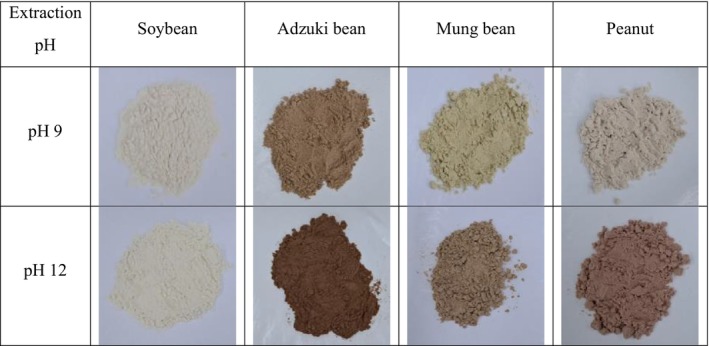
Physical appearance of legume protein concentrates at extraction pH 9 and 12.

The color differences and total polyphenol contents (TPC) at different protein extraction pH levels are listed in Table 2. The lightness (L*), redness (a*), yellowness (b*), and TPC of the legume proteins exhibited significant interaction effects between legume type and extraction pH (*p* < 0.001). The lightness ranged from 66.03 to 87.07 at pH 9 and 55.22 to 88.41 at pH 12. Soybean proteins exhibited higher lightness (87.07–88.41) than other legumes (55.22–81.10). Notably, lightness decreased at pH 12 compared to that at pH 9 for all legume proteins, except soybeans, resulting in visibly darker protein samples (Figure 1). Redness values ranged from 0.27 to 7.68 at pH 9 and from −1.29 to 10.40 at pH 12, whereas yellowness values ranged from 10.76 to 13.84 at pH 9 and from 7.54 to 14.54 at pH 12. Among the legumes, redness was highest in adzuki bean proteins (7.68–8.00), whereas mung beans tended to have the highest yellowness (14.34–19.72). Redness at pH 12 was higher than at pH 9 for legumes other than soybeans, whereas yellowness decreased. TPC ranged from 1.87 to 2.19 mg GAE/g at pH 9 and from 0.86 to 1.92 mg GAE/g at pH 12. Adzuki bean proteins exhibited lower TPC (0.86–1.39 mg GAE/g) than other legumes (1.56–2.19 mg GAE/g). Remarkably, TPC decreased at pH 12 compared to pH 9 in all legume proteins, with the exception of soybean.

**TABLE 2:** Effect of the extraction pH on color parameters and total polyphenol contents of different protein sources.

Legumes	Extraction pH	Lightness (L*)	Redness (a*)	Yellowness (b*)	Total polyphenol (mg GAE/g)
Soybean	pH 9	87.07 ± 0.57^a^	0.27 ± 0.14*	13.84 ± 0.53	1.90 ± 0.02
	pH 12	88.41 ± 0.56	−1.29 ± 0.19	14.54 ± 0.25	1.92 ± 0.00
Adzuki bean	pH 9	66.03 ± 0.21*	7.68 ± 0.02	10.76 ± 0.13**	1.39 ± 0.01**
	pH 12	55.22 ± 1.70	8.00 ± 0.04**	7.54 ± 0.40	0.86 ± 0.03
Mung bean	pH 9	77.17 ± 0.16*	2.54 ± 0.79	19.72 ± 0.24*	1.87 ± 0.01**
	pH 12	66.60 ± 1.50	5.74 ± 0.54*	14.34 ± 1.41	1.56 ± 0.02
Peanut	pH 9	81.10 ± 0.90**	2.83 ± 0.29	11.97 ± 0.30*	2.19 ± 0.01**
	pH 12	67.80 ± 0.10	10.4 ± 0.12***	10.4 ± 0.35	1.88 ± 0.03
Legumes (L)		***^b^	***	***	***
pH (P)		***	***	***	***
L × *P*		***	***	***	***

^a^ Significant differences between extraction conditions within the same protein source were determined using a *t*‐test (*p* < 0.05, *p* < 0.01, and *p* < 0.001 indicated by *, **, and ***, respectively).

^b^ Significant differences between the legume type and extraction pH were determined using a two‐way ANOVA test (*p* < 0.05, *p* < 0.01, and *p* < 0.001 indicated by *, **, and ***, respectively).

In this study, highly alkaline extraction conditions tended to reduce the lightness, yellowness and TPC and increase the redness of the extracted proteins. Due to their lighter color, soybean proteins are more suitable for food‐processing applications. Although protein extraction under highly alkaline conditions can reduce antinutritional factors such as phytates, it has the drawback of inducing protein browning (Jang and Yoon 2015). Protein browning occurs non‐enzymatically through the Maillard reaction, which involves the reaction of amino acids with amino side chains, such as lysine and reducing sugars (Deak and Johnson 2007; Jang and Yoon 2015). In this study, however, no significant changes in amino acid composition were observed for soybean and mung bean proteins (Table 3). Thus, the darker appearance of proteins under highly alkaline conditions was attributed to the conversion of polyphenols into quinones (Weemaes et al. 1998; Zagrean‐Tuza et al. 2020). Consistently, our results showed a decrease in TPC under highly alkaline extraction conditions, which can be explained by the quinone formation of polyphenols. Similarly, previous studies reported that quinones formation under highly alkaline conditions led to a decrease in TPC and antioxidant capacity in green and black tea (Doğan and Gökmen 2015).

**TABLE 3:** Effect of the extraction pH on amino acid composition (g/100 g) and digestibility (%) of proteins from different sources.

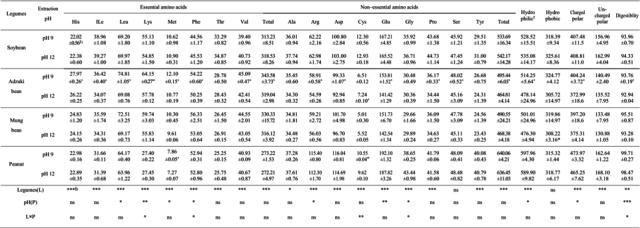

^a^ Hydrophilic (His, Lys, Thr, Arg, Asp, Glu, Cys, Ser, Tyr); Hydrophobic (Gly, Phe, Met, Ile, Leu, Val, Ala, Pro); Chared polar (His, Lys, Arg, Asp, Glu); Uncharged polar (Gly, Ser, Tyr, Cys, Thr).

^b^ Significant differences between extraction conditions within the same protein source were determined using a *t*‐test (*p* < 0.05, *p* < 0.01, *p* < 0.001 indicated by *, **, and ***, respectively).

^c^ Significant differences between the legume type and the extraction pH were determined using a two‐way ANOVA test (*p* < 0.05, *p* < 0.01, *p* < 0.001 indicated by *, **, and ***, respectively).

### 3.3 Particle Size Distribution

**FIGURE 2:**
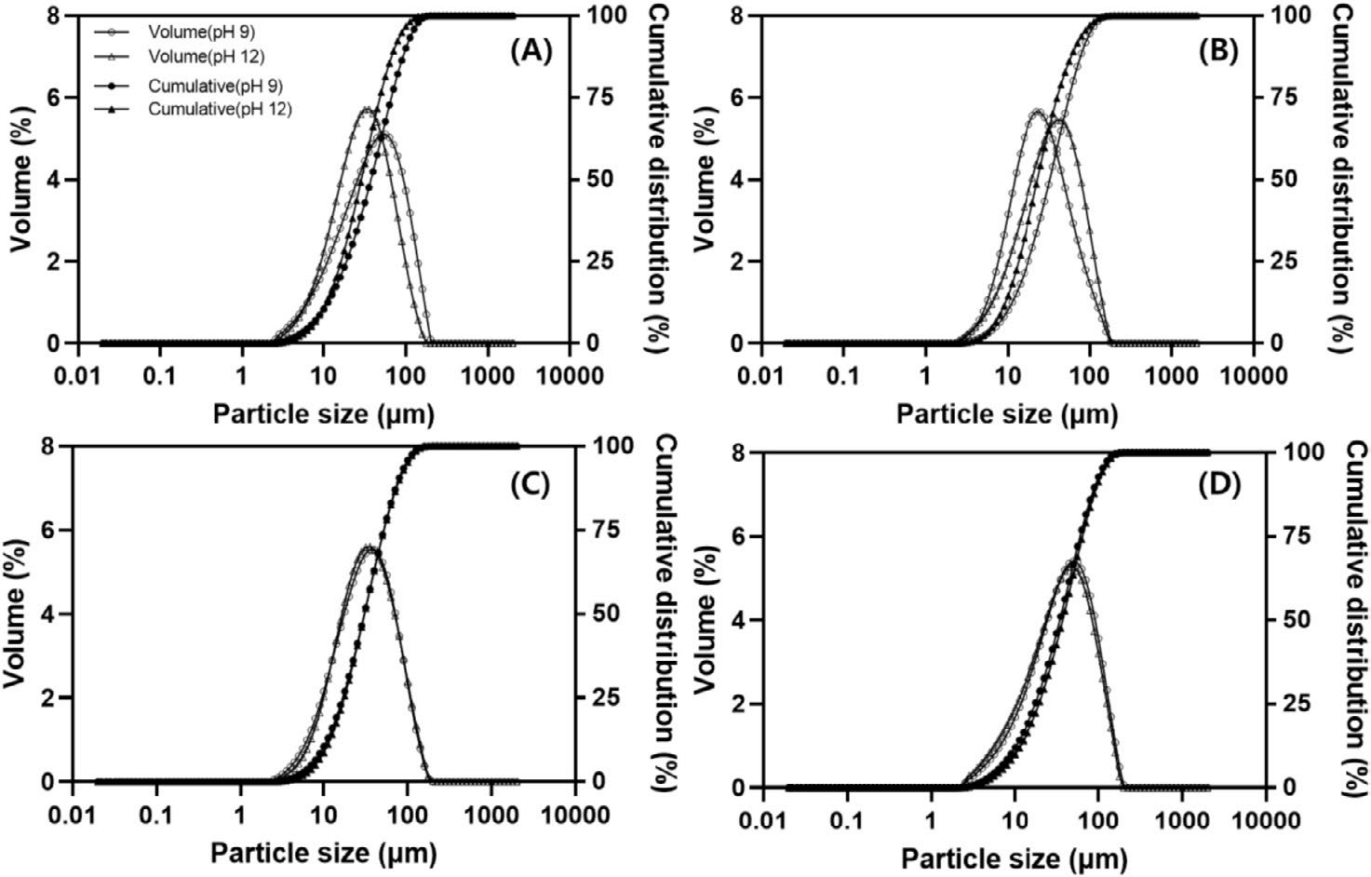
Particle size distribution of legume protein concentrates at extraction pH 9 and 12: (A) soybean, (B) adzuki bean, (C) mung bean, (D) peanut.

The particle size distributions of the proteins extracted at different pH levels are shown in Figure 2. The *d*(0,10) of legume proteins ranged from 10.07 to 10.95 μm at pH 9 and from 9.93 to 11.96 μm at pH 12. The *d*(0,50) ranged from 34.19 to 42.24 μm at pH 9 and from 25.87 to 38.92 μm at pH 12, whereas the *d*(0,90) ranged from 87.59 to 112.07 μm at pH 9 and from 73.87 to 101.58 μm at pH 12. The *D*[4,3] was 42.80–53.00 μm at pH 9 and 35.00–48.40 μm at pH 12. Notably, significant differences in d(0.90) were observed among legume types, with soybean (81.68–112.07 μm) and peanut proteins (101.58–107.36 μm) exhibiting relatively larger particles, whereas adzuki bean (73.87–93.80 μm) and mung bean proteins (87.59–89.37 μm) exhibited smaller ones. At pH 12, the *D*[4,3] of soybean, adzuki bean, and peanut proteins was 40.28, 35.02, and 48.34 μm, respectively, which were smaller than those at pH 9 (52.97, 45.60, and 51.82 μm, respectively).

Particle size is generally recognized as a critical physical property that influences various functional properties, including color, solubility, and foaming characteristics (Bakhsh et al. 2021). In our study, *D*[4,3] decreased under highly alkaline extraction conditions, particularly for soybean and adzuki bean proteins. Similarly, Abd Rahim et al. (2023) reported that the reduction in the particle size of rice bran protein was attributed to increased solubility caused by increased extraction pH. Conversely, in the present study, the solubilities of soybeans, adzuki beans, and peanuts significantly decreased under highly alkaline conditions (Table 5), suggesting that high ionic strength at pH 12 increased hydrophobic interactions within the proteins, thereby reducing particle size (Das et al. 2021). Conversely, Wu et al. (2021) observed that the particle size of tartary buckwheat protein increased as the extraction pH increased from 7 to 13, which they attributed to protein aggregation under highly alkaline conditions.

### 3.4 Constituent Amino Acid Composition and In Vitro Protein Digestibility

Amino acid compositions at different protein extraction pH levels are shown in Table 3. Lysine (*p* < 0.05), phenylalanine (*p* < 0.05), cysteine (*p* < 0.01), and glycine (*p* < 0.05) exhibited significant interaction effects between the legume type and extraction pH. Except for serine, the contents of all amino acids exhibited significant differences depending on the legume type. Among the essential amino acids, the contents of leucine and methionine varied significantly with the extraction pH (*p* < 0.05), exhibiting significantly lower values at pH 12 (68.05 ± 2.71, 9.64 ± 1.56 mg/g) compared to pH 9 (70.18 ± 4.52, 10.22 ± 1.68 mg/g), regardless of the legume type (*p* < 0.05). Furthermore, in adzuki bean proteins, essential amino acids except for threonine exhibited lower values at pH 12 than at pH 9; however, the differences were small. The total essential amino acid content of adzuki bean proteins was lower at pH 12 than at pH 9. Among the non‐essential amino acids, the contents of arginine (*p* < 0.05) and glutamic acid (*p* < 0.01) also varied significantly according to the legume type and extraction pH. Proteins extracted at pH 12 exhibited significantly lower arginine (71.49 ± 25.46 mg/g) and glutamic acid contents (159.36 ± 20.50 mg/g) compared to those extracted at pH 9 (73.94 ± 25.67, 166.23 ± 17.5 mg/g), regardless of the legume type (*p* < 0.05). Furthermore, in adzuki bean proteins, arginine, aspartic acid, glutamic acid, proline, and serine contents were lower at pH 12 than at pH 9, whereas cysteine content was higher at pH 12. Total non–essential amino acid content of adzuki bean protein was lower in proteins extracted at pH 12 than in those extracted at pH 9. Hydrophilic and charged polar amino acids exhibited significant differences depending on the extraction pH, with higher values observed at pH 12 (535.44 ± 41.57, 420.72 ± 34.18 mg/g) than those observed at pH 9 (519.85 ± 50.55, 405.59 ± 40.19 mg/g), regardless of the legume type (*p* < 0.05). Furthermore, the adzuki bean proteins exhibited significant differences in hydrophilicity (*p* < 0.05) and charged polar amino acids (*p* < 0.05) depending on the extraction pH. In vitro protein digestibility (IVPD) showed significant interaction effects between the legume type and extraction pH (*p* < 0.05). IVPD ranged from 93.76% to 99.71% at pH 9 and from 92.94% to 98.47% at pH 12, with peanuts (98.47%–99.71%) exhibiting a higher IVPD than proteins of other legumes (92.94%–95.51%). Within the same legume type, the IVPD was higher at pH 9 than at pH 12 for adzuki beans.

**FIGURE 3:**
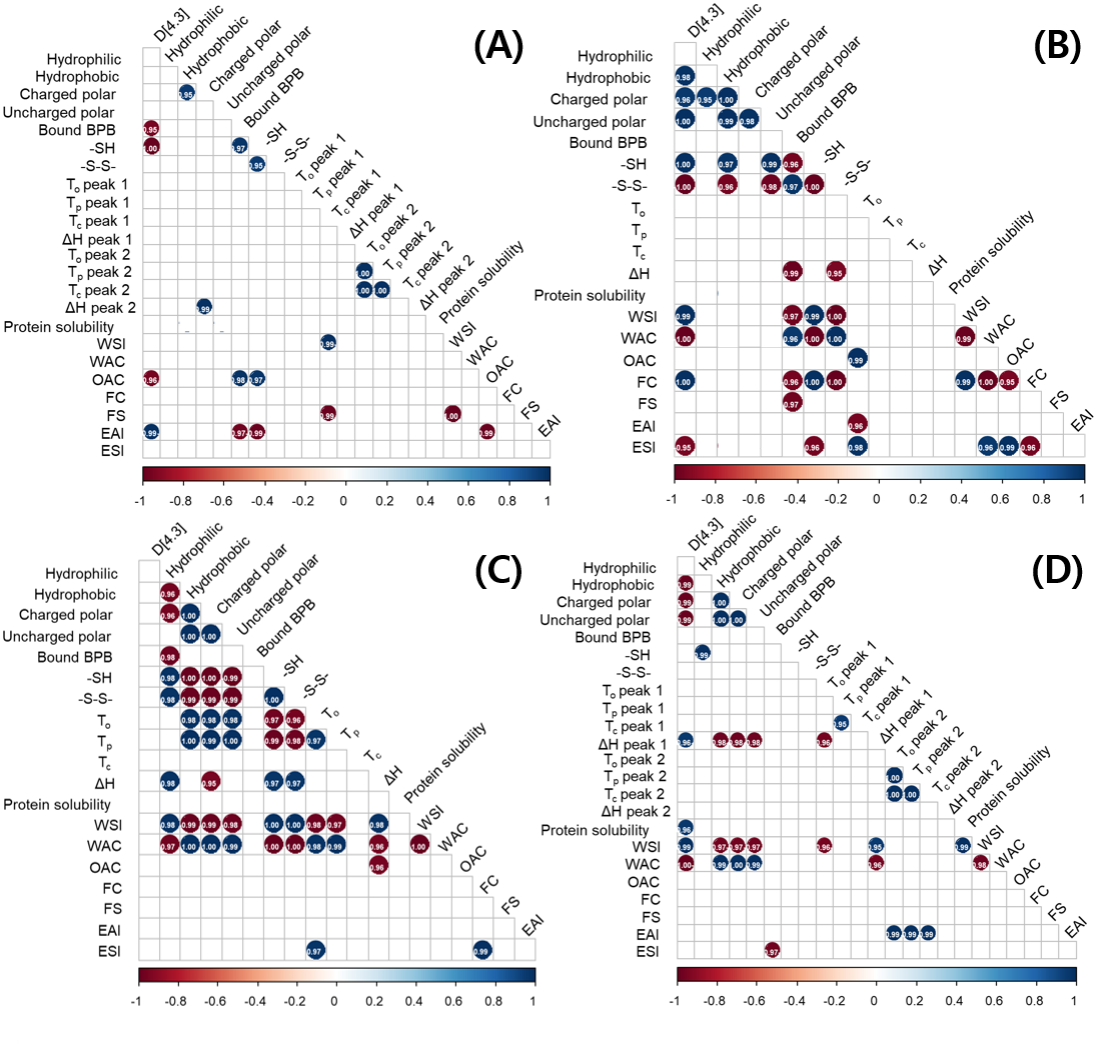
Correlation matrix among variables. In the matrix, red indicates a strong negative correlation, whereas blue signifies a strong positive correlation at *p* < 0.05. Blank spaces indicate non‐significant correlations. (A) Soybean, (B) adzuki bean, (C) mung bean, (D) peanut.

Kolpakova and Kovalenok (2019) reported that the solubility of wheat gluten showed a strong positive correlation with nonpolar amino acids and that its water absorption capacity was inversely correlated with polar amino acids, indicating that amino acid composition is related to the functional properties of proteins. Hadinoto et al. (2024) reported significant differences in alanine, leucine, threonine, proline, phenylalanine, glutamic acid, and lysine content in proteins extracted from the spent grain of brewers under pH 8–12 conditions. Kheto et al. (2024) also reported significant differences in the content of all amino acids, except glutamine, in proteins extracted from guar gum at pH 7–11. In this study, the differences in amino acid composition according to pH were likely due to differences in the crude protein content of the extracted adzuki bean proteins (Table 1) and the partial denaturation of certain amino acids under highly alkaline conditions. However, since no significant correlations were observed between hydrophilic, hydrophobic, polar, or non‐polar amino acids and the functional protein properties in soybean and adzuki bean (Figure 3), it can be implied that these differences had minimal impact on the functional properties of the proteins.

**FIGURE 4:**
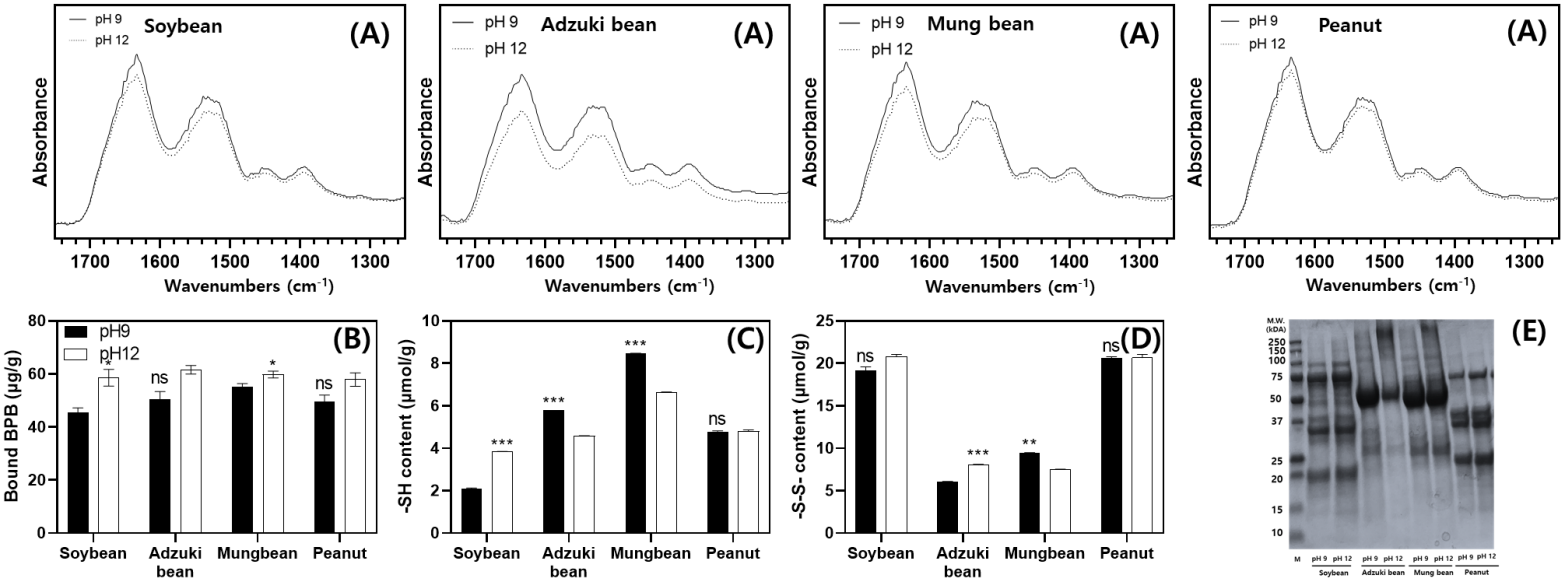
FTIR analysis, surface hydrophobicity, sulfhydryl group (—SSH), disulfide bonds (—SS—SS—S) contents, and SDS‐PAGE analysis of legume protein concentrates at extraction pH 9 and 12. (A) FTIR analysis, (B) surface hydrophobicity, (C) —SH content, (D) —SS—SS—S content, (E) SDS‐PAGE analysis. Significant differences between extraction conditions within the same protein source were determined using a *t*‐test (*p* < 0.05, *p* < 0.01, and *p* < 0.001 indicated by *, **, and ***, respectively).

The development of plant‐based alternative foods should take into account not only functional properties but also nutritional quality. In this study, a significant reduction in leucine, methionine, arginine, and glutamic acid contents was observed under highly alkaline conditions, regardless of legume type. These results suggest that highly alkaline conditions can induce racemization and promote the formation of lysinoalanine, both of which have been reported to negatively affect protein digestibility (Gilani et al. 2005; Guan et al. 2017; Bellagamba et al. 2015; Zhang et al. 2018). A decrease in protein digestibility was particularly evident in adzuki bean, where the amino acid composition was markedly altered, with a difference of 0.82 percentage points. Carbonaro et al. (1997) reported that heat‐induced protein aggregation reduced solubility, thereby limiting enzyme accessibility and ultimately decreasing digestibility. Consistently, in our study, extraction under highly alkaline conditions induced aggregation of adzuki bean proteins (Figure 4E), reduced their solubility (Table 5), and consequently decreased IVPD. Furthermore, because the calculation of IVPD involves a correction of amine group concentration based on proline, lysine, histidine, and arginine (Pinel et al. 2025), changes in the amino acid composition of adzuki bean proteins caused by highly alkaline extraction also contributed to altered IVPD values. In addition, lysinoalanine has been reported to increase in NaOH‐treated proteins but subsequently decrease due to degradation (Hou et al. 2017), while Zhang et al. (2018) demonstrated that cysteine, lysine, threonine, and arginine are degraded under highly alkaline conditions to form lysinoalanine in rice residue. Therefore, further studies are required to clarify the effects of lysinoalanine‐containing proteins not only on nutritional quality and digestibility but also on functional properties.

### 3.5 Protein Structural Analysis

#### 3.5.1 Secondary Structure

The FTIR spectra of proteins derived from various legume sources are presented in Figure 4A. Analysis revealed distinct absorption bands at 1480–1575 cm^−1^ and 1600–1690 cm^−1^, which are attributed to the amide I and amide II regions, respectively. In addition, the proteins of all legumes exhibited lower absorbance values at pH 12 than at pH 9, with this behavior being more pronounced in adzuki bean and mung bean. Notably, adzuki bean showed a particularly distinct behavior in the 1229–1301 cm^−1^ region corresponding to the amide III band.

Shevkani et al. (2019) reported that the secondary structure of proteins influences their behavior during food processing. Amide I (C=O stretching) represents the most sensitive spectral region for elucidating protein secondary structures, whereas amide II (N—H bending coupled with C—N stretching) is primarily associated with backbone vibrations, and amide III arises from mixed structural contributions, including potential interactions with carbohydrates (Shrestha et al. 2023). In this study, the absorbance at pH 12 was generally lower than that at pH 9, indicating a partial loss of secondary structures, specifically a decrease in α‐helix and β‐turns, and a possible increase in random coil content under highly alkaline conditions. Consistent with these observations, Jarpa‐Parra et al. (2014) also reported in lentil proteins that elevating extraction pH promotes partial unfolding/denaturation by disrupting hydrogen bonding and enhancing the deprotonation of ionizable groups (e.g., carboxylate/sulfate), thereby increasing surface charge. Meanwhile, Zhaoli Zhang et al. (2018) reported that, as NaOH concentration increased, rice residue protein isolates exhibited unfolding of α‐helices and β‐turns with a concomitant increase in β‐sheet content, whereas lysinoalanine‐containing protein showed decreases in β‐sheet/β‐turn and increases in α‐helix and random coil, indicating that alkaline treatment readily disrupts noncovalent and covalent interactions, resulting in partial denaturation and structural rearrangement.

#### 3.5.2 Tertiary Structure

The surface hydrophobicity tended to be higher at pH 12 (59.47 ± 2.28 μg/g) than at pH 9 (50.12 ± 4.09 μg/g). Specifically, the surface hydrophobicity was 1.08 times higher for soybean proteins and 1.22 times higher for mung bean proteins at pH 12 than at pH 9 within the same legume type (Figure 4B). The sulfhydryl group (—SH) and disulfide bond (—S—S—) content exhibited different trends depending on the legume type and the extraction pH (Figure 4C,D). In soybean proteins, the –SH content at pH 12 was 1.82 times higher than that at pH 9, whereas in adzuki bean and mung bean proteins, the —SH content at pH 9 was 1.27 and 1.26 times higher, respectively. In the adzuki bean proteins, the —S—S— content at pH 12 was higher than that at pH 9, whereas in the mung bean proteins, the —S—S— content was higher at pH 9.

Surface hydrophobicity changes as hydrophobic regions previously buried within the core of globular proteins are exposed to the surface owing to protein denaturation (Schwenke 2001). In this study, the surface hydrophobicity tended to increase under highly alkaline extraction conditions, aligning with the findings of Jiang et al. (2018). Jiang et al. (2018) reported that during the refolding of structurally unfolded proteins returning to neutral pH under extreme pH conditions, molten globules were formed, increasing surface hydrophobicity. The —SH and —S—S— contents play essential roles in stabilizing the structural conformation of proteins and are critical for their functional properties (Li et al. 2019). The —SH content can change because of protein denaturation, which exposes the —SH, or because of the disruption and formation of disulfide bonds caused by the dissociation of protein subunits (Li et al. 2019). In this study, the —SH content of soybean proteins increased under high‐alkaline extraction conditions due to exposure, whereas it decreased in adzuki bean and mung bean proteins, likely due to the formation of disulfide bonds. Furthermore, the differences in —SH content among legume types were attributed to variations in amino acid composition, with soybean and peanut proteins having higher cysteine content and forming more disulfide bonds than mung bean and adzuki bean proteins.

#### 3.5.3 SDS‐PAGE

Figure 4A shows the SDS–PAGE profiles of proteins extracted at different pH values across the four legume types. The subunit banding patterns were essentially identical regardless of extraction pH in soybean and peanut proteins, indicating that alkaline extraction did not affect subunit composition. In contrast, adzuki bean and mung bean proteins displayed noticeable differences depending on the extraction pH. Specifically, an additional band above ~250 kDa was detected under highly alkaline conditions, suggesting the formation of high‐molecular‐weight aggregates. In adzuki bean proteins, the marked reduction in the 50 kDa band intensity further suggests that specific subunits, likely corresponding to vicilin‐like (7S), were destabilized and either degraded or incorporated into aggregate complexes under highly alkaline extraction (Philadelpho et al. 2021). This observation is consistent with previous reports indicating that highly alkaline extraction can cause partial hydrolysis into lower molecular weight fractions, while simultaneously promoting hydrophobic interactions and intermolecular disulfide bond formation that drive the assembly of insoluble aggregates subsequently removed during centrifugation (Wang et al. 2022; Ruiz et al. 2016). In conclusion, these results imply that, unlike soybean and peanut proteins, adzuki bean and mung bean proteins are more susceptible to structural rearrangements and aggregation when exposed to highly alkaline environments.

### 3.6 Thermal Properties

**FIGURE 5:**
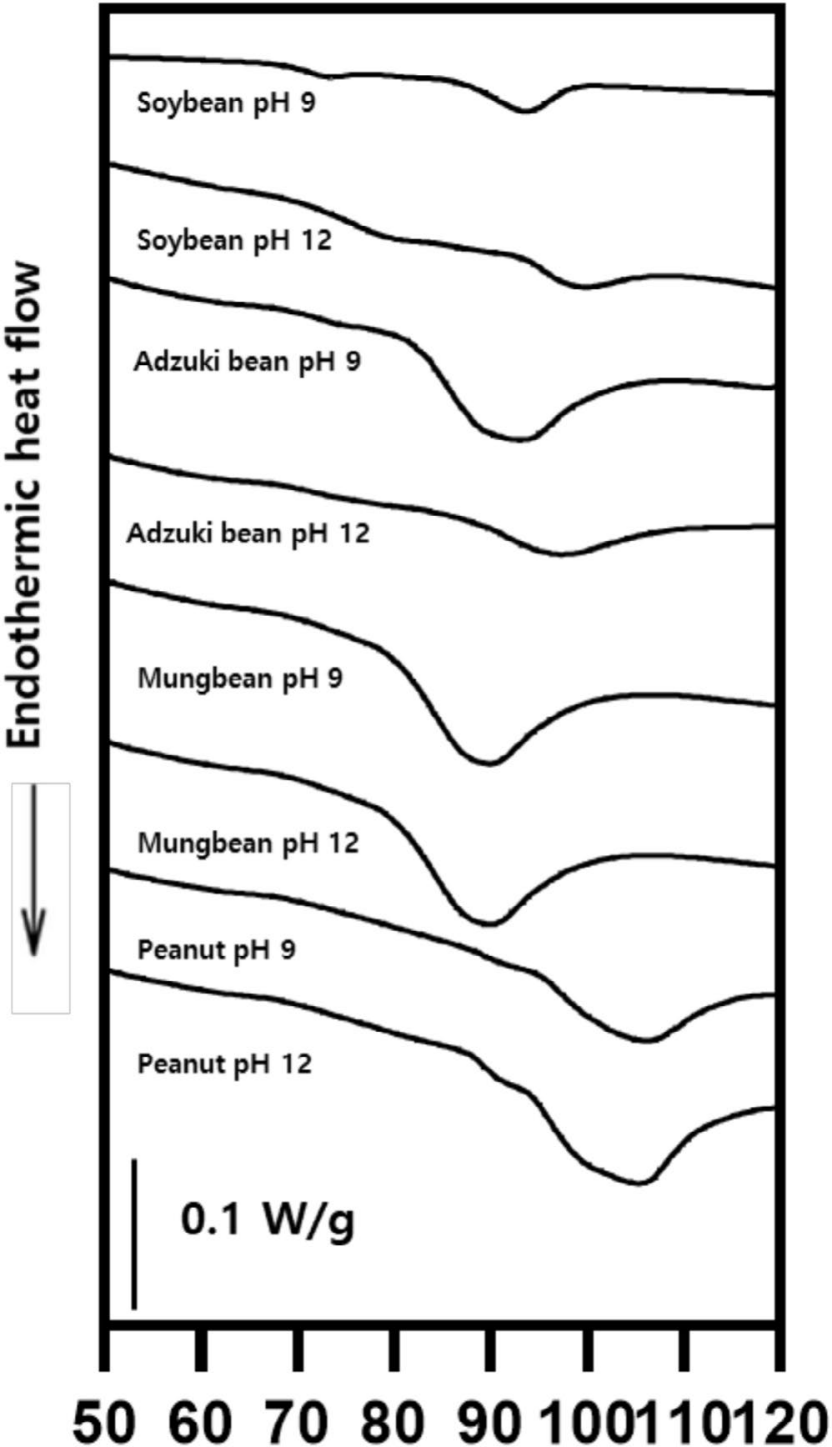
Thermal properties of legume proteins at extraction pH 9 and 12.

The thermal properties of the legume proteins are presented in Table 4. Soybean and peanut proteins exhibited a double peak, whereas adzuki bean and mung bean proteins showed a single peak, indicating differences among the legume types (Figure 5). The onset temperature (*T*_o_) of mung bean proteins extracted at pH 12 was significantly higher than that extracted at pH 9 by 5.32°C. Similarly, the peak temperature (*T*_p_) of mung bean proteins extracted at pH 12 was higher by 6.83°C than that at pH 9, whereas the *T*_p_ of adzuki bean proteins was higher at pH 12 by 3.08°C than at pH 9. The denaturation enthalpy (ΔH) showed a significant difference depending on the extraction pH (*p* < 0.05), with proteins extracted at pH 12 (0.89 ± 1.07 J/g) being significantly lower than those extracted at pH 9 (2.09 ± 2.27 J/g), regardless of the legume type (*p* < 0.05). In soybean proteins, the ΔH of peak 1 was 0.02 J/g lower at pH 12 than at pH 9.

**TABLE 4:** Effect of the extraction pH on thermal properties of different protein sources.

Legumes	Peak	Extraction pH	*T*_o_	*T*_p_	*T*_c_	ΔH
Soybean	Peak1	pH 9	73.02 ± 6.06^a^	76.89 ± 5.87	81.04 ± 6.70	0.34 ± 0.17*
		pH 12	73.19 ± 3.52	79.33 ± 0.81	86.60 ± 4.24	0.32 ± 0.03
	Peak2	pH 9	90.59 ± 4.69	96.99 ± 5.00	102.54 ± 5.83	2.28 ± 0.91
		pH 12	94.68 ± 1.49	99.73 ± 0.66	108.45 ± 2.04	0.83 ± 0.05
Adzuki bean	Peak1	pH 9	80.77 ± 1.45	90.17 ± 0.84	100.05 ± 2.03	4.41 ± 1.2
		pH 12	88.59 ± 2.98	97.00 ± 0.38**	106.62 ± 2.33	1.58 ± 0.72
Mung bean	Peak1	pH 9	79.88 ± 0.21	88.68 ± 0.49	97.32 ± 1.72	4.29 ± 0.80
		pH 12	85.20 ± 1.32*	91.76 ± 0.08*	98.08 ± 0.28	1.21 ± 0.33
Peanut	Peak1	pH 9	88.35 ± 0.30	90.66 ± 0.07	94.12 ± 0.77	0.09 ± 0.02
		pH 12	88.21 ± 0.37	91.21 ± 0.66	94.54 ± 0.71	0.05 ± 0.01
	Peak2	pH 9	92.68 ± 1.17	104.41 ± 0.39	112.00 ± 0.10	4.78 ± 0.44
		pH 12	95.68 ± 0.41	105.47 ± 0.83	113.21 ± 1.46	2.84 ± 0.26
Legumes (L)			ns^b^	ns	ns	ns
pH (P)			ns	ns	ns	*
L × *P*			ns	ns	ns	ns

Abbreviations: *T*_c_, Conclusion temperature; *T*_o_, Onset temperature; *T*_p_, Peak temperature; ΔH, Denaturation enthalpy.

^a^ Significant differences between extraction conditions within the same protein source were determined using a *t*‐test (*p* < 0.05, *p* < 0.01, and *p* < 0.001 indicated by *, **, and ***, respectively).

^b^ Significant differences between legumes and extraction pH were determined using a two‐way ANOVA test (*p* < 0.05, *p* < 0.01, and *p* < 0.001 indicated by *, **, and ***, respectively).

The thermal properties of proteins are important for cooking, drying, and sterilization. In this study, proteins extracted under highly alkaline conditions displayed higher *T*_o_ and *T*_p_ values than those extracted at pH 9, indicating that a higher temperature was required to initiate and reach the maximum rate of unfolding. The increase in *T*_o_ and *T*_p_ of adzuki bean and mung bean proteins at pH 12 was likely due to the formation of stable protein aggregates as the denatured proteins were reassembled (Tang et al. 2007). Furthermore, the ΔH tended to decrease, particularly with peak 1 of soybean proteins. The ΔH represents the amount of energy required for protein denaturation, reflecting the net energy needed to break hydrogen bonds (endothermic) and hydrophobic interactions (exothermic) (Brishti et al. 2020). Jiang et al. (2010) reported that soybean protein structures (7S and 11S) extracted at pH 12 extensively unfolded, weakening the intramolecular bonds determining the thermal properties. They attributed the reduction in ΔH to the collapse of tertiary structures rather than secondary structures. In a study examining the effects of alkaline extraction on peanut proteins, Liu et al. (2011) observed that protein denaturation occurred even under mildly alkaline conditions (pH 8), with 11S being more susceptible to pH‐induced changes than 7S, leading to decreased thermal stability. Voutsinas et al. (1983) reported a correlation between thermal properties (such as protein coagulation and gelation) and surface hydrophobicity. However, in this study, no significant correlation was found between surface hydrophobicity and thermal properties, such as the ΔH of Peak 1 in soybean proteins, the *T*_p_ of adzuki bean proteins, and the *T*_o_ and *T*_p_ of mung bean proteins, which varied depending on the extraction pH (Figure 3). In conclusion, proteins extracted under highly alkaline conditions exhibited higher *T*_o_ and *T*_p_ values, indicative of greater apparent thermal resistance, while their ΔH was reduced. This suggests that proteins extracted at highly alkaline conditions undergo partial unfolding followed by aggregation, which reinforces the structural network and shifts the thermal transition to higher temperatures, while simultaneously reducing the number of intact native domains available for cooperative unfolding, thereby lowering the overall ΔH.

### 3.7 Functional Properties

#### 3.7.1 Water‐ and Oil‐Related Properties

The solubility of legume proteins showed significant differences among legume types (*p* < 0.05), with peanut proteins (33.94% ± 1.03%) exhibiting significantly higher solubility than mung bean proteins (28.99% ± 0.71%), regardless of extraction pH (Table 5) (*p* < 0.05). The extraction pH also significantly affected the solubility (*p* < 0.01), with a significantly lower value observed at pH 12 (29.73% ± 2.45%) compared to that recorded at pH 9 (33.15% ± 2.69%), regardless of the legume type (*p* < 0.05). The water solubility index (WSI) and water absorption capacity (WAC) of legume proteins showed a significant interaction effect between the legume type and the extraction pH (*p* < 0.001), whereas the oil absorption capacity (OAC) showed a significant effect at *p* < 0.05. The WSI ranged from 87.90% to 92.69% at pH 9 and from 33.1% to 91.3% at pH 12. Notably, at pH 12, the WSI exhibited the highest value in soybean proteins, followed by peanut, mung, and adzuki bean proteins. Additionally, the WSI of the proteins extracted from adzuki beans, mung beans, and peanuts at pH 9 was 2.80, 1.78, and 1.25 times, respectively, higher than that of proteins extracted at pH 12. The WAC ranged from 0.75 to 1.35 g/g at pH 9 and from 1.48 to 16.6 g/g at pH 12. Notably, at pH 12, WAC was the highest in adzuki bean proteins, followed by mung bean, peanut, and soybean proteins. Furthermore, the WAC of proteins extracted from adzuki bean, mung bean, and peanut proteins at pH 12 was 22.13‐, 13.0, and 2.78 times higher than those extracted at pH 9. The OAC ranged from 2.30 to 2.49 g/g at pH 9 and from 2.23 to 2.66 g/g at pH 12. Soybean proteins extracted at pH 12 exhibited a higher OAC than those extracted at pH 9.

**TABLE 5:** Effect of extraction pH on water‐ and oil‐related functional properties of different protein sources.

Legumes	Extraction pH	Protein solubility (%)	WSI (%)	WAC (g/g)	OAC (g/g)
Soybean	pH 9	33.61 ± 0.83^a^	88.52 ± 2.64	1.35 ± 0.05	2.49 ± 0.01
	pH 12	28.77 ± 3.22	91.32 ± 1.48	1.48 ± 0.47	2.66 ± 0.04*
Adzuki bean	pH 9	34.82 ± 3.26	92.69 ± 0.02**	0.75 ± 0.01	2.33 ± 0.02
	pH 12	28.50 ± 0.72	33.10 ± 7.02	16.61 ± 0.13***	2.54 ± 0.07
Mung bean	pH 9	29.42 ± 0.42	87.90 ± 2.58**	0.90 ± 0.04	2.30 ± 0.13
	pH 12	28.56 ± 0.79	49.40 ± 1.94	11.72 ± 0.29***	2.52 ± 0.03
Peanut	pH 9	34.78 ± 0.15*	90.33 ± 1.43*	1.09 ± 0.04	2.30 ± 0.02
	pH 12	33.09 ± 0.53	72.19 ± 2.81	3.03 ± 0.01***	2.23 ± 0.04
Legumes (L)		*^b^	***	***	***
pH (P)		**	***	***	**
L × *P*		ns	***	***	*

Abbreviations: OAC, oil absorption capacity; WAC, water absorption capacity; WSI, water solubility index.

^a^ Significant differences between extraction conditions within the same protein source were determined using a *t*‐test (*p* < 0.05, *p* < 0.01, and *p* < 0.001 indicated by *, **, and ***, respectively).

^b^ Significant differences between the legume type and the extraction pH were determined using a two‐way ANOVA test (*p* < 0.05, *p* < 0.01, and *p* < 0.001 indicated by *, **, and ***, respectively).

Protein solubility is determined by the hydrophilic interactions between proteins and the solvent, hydrophobic interactions between proteins, and factors such as pore size (Xu et al. 2023). This study showed decreased solubility and WSI of proteins extracted under highly alkaline conditions. Gao et al. (2020) reported a decrease in the solubility of pea protein when the extraction pH increased from 8.5 to 9.5, which was attributed to increased protein aggregation, as evidenced by the increase in protein fractions larger than 2000 kDa at pH 9.5. Mundi and Aluko (2012) found that higher SH content in kidney bean proteins improved protein solubility. Similarly, this study showed a highly positive correlation between the WSI and SH content for adzuki (*r* = 0.993*) and mung (*r* = 0.997**) bean proteins, showing consistent results (Figure 3). WAC refers to the ability of proteins to retain water without dissolving, which plays a crucial role in reducing water loss and maintaining moisture and freshness in high‐viscosity products, such as soups, doughs, and baked goods. Ge et al. (2021) reported that the WAC of various legumes extracted at pH 10 ranged from 1.48 to 4.49 g/g, with soybean proteins exhibiting superior WAC compared to adzuki bean and mung bean proteins, partially aligning with the findings of this study. The significantly higher WAC of proteins extracted from adzuki beans, mung beans, and peanuts at pH 12 observed in this study may be due to the increased proportion of insoluble proteins caused by low solubility during the washing process, which in turn enhances water‐binding capacity (López et al. 2018). Furthermore, this suggests the need for further analysis of the hydrophilic carbohydrate and fiber content in each extracted protein. Additionally, the remarkably high WAC of adzuki bean and mung bean proteins extracted at pH 12 compared to previously reported values may be attributed to cultivar characteristics. In conclusion, adzuki and mung bean proteins extracted at pH 12 appear to be suitable for high‐viscosity products, whereas the water‐related properties of soybeans seem to be less influenced by the extraction pH. The oil absorption capacity, which is associated with mouthfeel and flavor retention in food, was likely higher for soybean proteins extracted at pH 12 because of their lower solubility and higher surface hydrophobicity, resulting in greater oil retention (Güzel et al. 2019).

#### 3.7.2 Foaming and Emulsifying Properties

The foaming capacity (FC) of legume proteins was significantly influenced by the interaction between the legume type and the extraction pH (*p* < 0.05) (Table 6). The FC ranged from 151.25% to 181.25% at pH 9 and from 105.00% to 213.75% at pH 12, with soybean proteins exhibiting the highest FC and peanut proteins the lowest. Moreover, the mung bean proteins extracted at pH 12 had a higher FC than those extracted at pH 9. Foam stability (FS) was also significantly affected by the interaction between the legume type and the extraction pH (*p* < 0.05). The FS ranged from 21.46% to 41.29% at pH 9 and from 25.68% to 37.30% at pH 12, with peanut proteins showing relatively lower values than proteins of other legumes. However, no significant differences in the extraction pH were observed for all legume types. The emulsifying activity index (EAI) differed significantly among legume types (*p* < 0.001). Regardless of the extraction pH, soybean (35.79 ± 3.25), adzuki bean (32.32 ± 4.40), mung bean (21.02 ± 1.21), and peanut (11.88 ± 0.60) proteins exhibited decreasing values in that order (*p* < 0.05). The extraction pH also significantly affected the EAI (*p* < 0.01), with a significantly lower value at pH 12 (23.78 ± 8.89) compared to that at pH 9 (26.72 ± 11.58) (*p* < 0.05), irrespective of the legume type. The emulsifying stability index (ESI) was significantly influenced by the interaction between the legume type and the extraction pH (*p* < 0.001). The ESI ranged from 42.88% to 56.61% at pH 9 and from 19.62% to 79.91% at pH 12, with mung bean proteins showing the highest values and peanut proteins the lowest. Furthermore, adzuki bean proteins extracted at pH 12 exhibited a higher ESI compared to those extracted at pH 9.

**TABLE 6:** Effect of the extraction pH on foaming‐ and emulsifying‐related functional properties of different protein sources.

Legumes	Extraction pH	FC (%)	FS (%)	EAI (m^2^/g)	ESI (%)
Soybean	pH 9	181.25 ± 18.75^a^	41.29 ± 3.60	38.58 ± 0.37**	45.49 ± 0.47
	pH 12	213.75 ± 18.75	37.30 ± 1.98	33.00 ± 0.68	51.94 ± 8.26
Adzuki bean	pH 9	157.50 ± 5.00	33.33 ± 2.24	35.29 ± 2.95	47.57 ± 0.20
	pH 12	145.00 ± 7.00	27.58 ± 2.43	29.34 ± 3.73	61.78 ± 4.10*
Mung bean	pH 9	151.25 ± 1.25	21.46 ± 3.25	21.08 ± 0.61	56.61 ± 1.29
	pH 12	197.50 ± 22.50**	32.98 ± 4.86	20.96 ± 2.00	79.91 ± 11.4
Peanut	pH 9	95.00 ± 5.00	25.00 ± 0.00	11.94 ± 0.65	42.88 ± 2.99
	pH 12	105.00 ± 7.50	25.68 ± 1.75	11.82 ± 0.81	19.62 ± 12.60
Legumes (L)		***^b^	***	***	***
pH (P)		**	ns	**	ns
L × *P*		*	*	ns	***

*Note:* Test (*p* < 0.05, *p* < 0.01, and *p* < 0.001 indicated by *, **, and ***, respectively).

Abbreviations: EAI, Emulsifying activity index; ESI, emulsification stability index; FC, Foaming capacity; FS, Foaming stability.

^a^ Significant differences between extraction conditions within the same protein source were determined using a *t*‐test (*p* < 0.05, *p* < 0.01, and *p* < 0.001 indicated by *, **, and ***, respectively).

^b^ Significant differences between the legume type and the extraction pH were determined using a two‐way ANOVA.

The foaming properties of proteins are important in applications that require soft and voluminous textures, such as cakes and whipped creams, whereas the emulsifying properties are critical for emulsified products, such as mayonnaise. Proteins contribute to foaming and emulsifying capacity by forming a physical barrier at the water‐air and water–oil interfaces, reducing interfacial tension and preventing coalescence. Additionally, proteins enhance the foam and emulsion stability by increasing the viscosity of the continuous phase (Sy 1993). Solubility and surface hydrophobicity are key factors influencing the foaming and emulsifying properties of proteins. High solubility increases the amount of protein that can migrate to the interface, whereas high surface hydrophobicity promotes protein adsorption at the interface, thereby enhancing these properties (Cui et al. 2020). In this study, foaming and emulsifying properties exhibited different trends depending on the legume type under high‐alkaline extraction conditions. For FC, all legume proteins except adzuki bean proteins showed higher values under high‐alkaline extraction conditions; however, significant differences were observed for only mung bean proteins. This may be attributed to the higher surface hydrophobicity of the mung bean proteins at pH 12, which is consistent with the findings of and Bae and Rhee (1998) for soybeans. Additionally, despite the lower values of the EAI under highly alkaline extraction conditions, only soybean proteins exhibited significant differences, likely due to their lower solubility at pH 12 (Table 5). Although the ESI tended to be higher under highly alkaline conditions for all legumes except peanuts, significant differences were observed only for adzuki bean proteins. This may be due to the negative correlation between the ESI of adzuki bean proteins and their mean particle size (*r* = −0.952*), indicating that smaller particles increased the viscosity of the continuous phase (Figure 3). In conclusion, proteins extracted at pH 12 exhibited superior FC, whereas those extracted at pH 9 demonstrated higher ESI, suggesting that proteins extracted at pH 12 are suitable for voluminous products, whereas those extracted at pH 9 are more appropriate for emulsified products. Moreover, soybean, adzuki bean, and mung bean proteins show better foaming and emulsifying properties than peanut proteins, suggesting their suitability as ingredients for plant‐based products, such as baked goods and sauces.

## 4 Conclusion

We investigated the effects of the extraction pH on the physicochemical, structural, and functional properties of legume proteins. The results revealed that highly alkaline extraction conditions (pH 12) generally enhanced the protein extraction yield while reducing color lightness. The amino acid composition of adzuki bean proteins showed significant changes depending on the protein extraction pH, whereas the particle size exhibited notable changes in soybean and adzuki bean proteins. The higher water absorption capacity observed in adzuki and mung bean proteins extracted at pH 12 highlights their potential for high‐viscosity applications such as soups and baked goods. Proteins extracted at pH 12 exhibited smaller particle sizes and higher surface hydrophobicity, contributing to their superior foaming capacity and making them suitable for applications requiring voluminous textures, such as baked goods and whipped creams. However, the emulsifying activity index was generally higher at pH 9, suggesting that proteins extracted under mildly alkaline conditions are more appropriate for emulsified products, such as mayonnaise and dressings. Furthermore, soybean, adzuki bean, and mung bean proteins demonstrate superior foaming and emulsifying properties compared to peanut proteins, making them promising candidates for plant‐based products.

## Author Contributions

**Hyun‐Jin Park:** writing – original draft (lead). **You‐Geun Oh:** conceptualization (supporting). **Areum Chun:** supervision (equal). **Jung Hyun Seo:** resources (supporting). **Eunyoung Oh:** resources (supporting). **Ji Ho Chu:** resources (supporting). **Young Kwang Ju:** resources (supporting). **Sang‐Jin Ye:** supervision (equal).

## Ethics Statement

This study does not involve any human or animal testing.

## Conflicts of Interest

The authors declare no conflicts of interest.

## Data Availability

The datasets generated and/or analyzed during the current study can be obtained from the corresponding author upon reasonable request.
